# Heat-Passing Framework for Robust Interpretation of Data in Networks

**DOI:** 10.1371/journal.pone.0116121

**Published:** 2015-02-10

**Authors:** Yi Fang, Mengtian Sun, Karthik Ramani

**Affiliations:** 1 Electrical and Computer Engineering, New York University Abu Dhabi, Abu Dhabi, UAE; 2 School of Mechanical Engineering, Purdue University, West Lafayette, Indiana, United States of America; Universitat Pompeu Fabra, Barcelona Research Park of Biomedicine (PRBB), SPAIN

## Abstract

Researchers are regularly interested in interpreting the multipartite structure of data entities according to their functional relationships. Data is often heterogeneous with intricately hidden inner structure. With limited prior knowledge, researchers are likely to confront the problem of transforming this data into knowledge. We develop a new framework, called *heat-passing*, which exploits intrinsic similarity relationships within noisy and incomplete raw data, and constructs a meaningful map of the data. The proposed framework is able to rank, cluster, and visualize the data all at once. The novelty of this framework is derived from an analogy between the process of data interpretation and that of heat transfer, in which all data points contribute simultaneously and globally to reveal intrinsic similarities between regions of data, meaningful coordinates for embedding the data, and exemplar data points that lie at optimal positions for heat transfer. We demonstrate the effectiveness of the heat-passing framework for robustly partitioning the complex networks, analyzing the globin family of proteins and determining conformational states of macromolecules in the presence of high levels of noise. The results indicate that the methodology is able to reveal functionally consistent relationships in a robust fashion with no reference to prior knowledge. The heat-passing framework is very general and has the potential for applications to a broad range of research fields, for example, biological networks, social networks and semantic analysis of documents.

## Introduction

Advances in information technologies coupled with new data generation sources have resulted in the production of data at an unprecedented scale from sources as diverse as social networks, web pages, protein sequences and multimedia images. Given the lack of sufficient prior knowledge about the data, it is often challenging for people to infer a clear picture of the internal mechanism through which regions of the data set interact meaningfully with each other. An effective way of interpreting data is to place all of the data in a network and to study the behavior of the network system governed by agreement among all individual interactions. The measured behavior can now robustly reflect the hidden structure behind the network [1, 2]. The advantage of placing data in a network has been highlighted by the commercial success of Google, attributed to the elegant algorithm PageRank, which exploits global structure of cyber network from the local hyper-link of the web-page [3]. PageRank characterizes the behavior of the web-pages’ network through a probability distribution of the resulting random walk mapped onto the network. The innovation of PageRank has inspired data mining procedures for scientists in biological and social science, for example, protein ranking and community structure detection [1, 4]. Practically, the superiority of structure-based approaches arises from the process of propagating the local similarity to global information that redefines the similarity among any data pair [5, 6]. That is, after propagation of local similarity, the relationship among the data points is redefined according to the re-organization of all of the data points simultaneously. This map retains a great deal of important information, provides a concise picture of the interactions within the data, and highlights underlying intrinsic structures of the data set.

In this paper, we emphasize the advantage gained by exploiting the global intrinsic structure behind the data as encoded in the *geometric heat flow* on the network, and develop the method to interpret the revealed structure. We assume that all of the data can be properly embedded in a manifold where the original local interactions (distance or similarity) are approximately preserved [7]. We then use heat flow as a proxy for information flow over the network. The advantage thereby is that heat is sensitive to the geometric structure of the data, and its flow over the network intrinsically takes all local infinitesimal interactions of a system into account [8, 9]. In short, our point of view is that to understand the network, we need to understand the system behavior of the network reflected by the flow of heat on it. The *global* rate of heat transfer across the entire network depends on the points of application of heat. This observation allows us to identify a number of nodes with high overall tendency to transfer heat to all other points in an efficient manner. This identification is carried out through an analogy between heat passing and *message passing*, and ensures the global optimal determination of the nodes of application of heat, which we call heat centers [10, 11]. Using the framework we developed, we demonstrate that the heat-passing is able to complete the tasks of data ranking, visualizing, and clustering in our heat featured space all at once (refer to the section ‘Method’ below).

**Figure 1:**
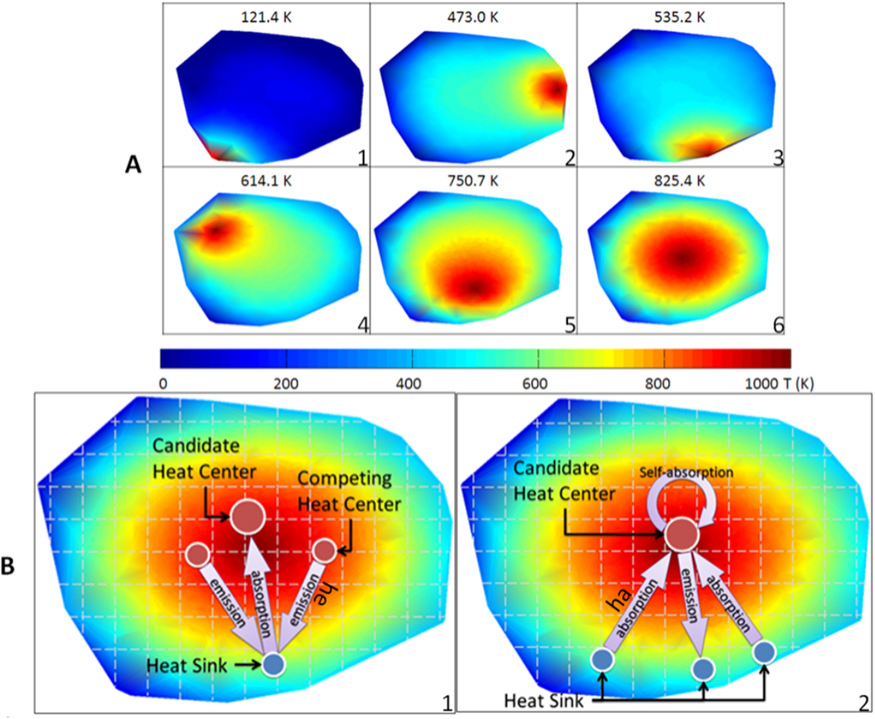
Illustration of heat flow and heat passing. Heat-passing framework is to understand the system behavior of a network reflected by the flow of heat over it. This figure illustrates the heat flow across the network by applying the heat source on different nodes in (A) and the message exchange of heat passing process in (B). In (A), there are six figures displaying the temperature distribution after heat flows for a certain time with application of the heat source on six different nodes. The normalized temperature is listed on the top of each figure. The average temperature is used to initialize the clustering center preference value *hcpv*. The higher the temperature the faster the heat flows on the network. In (B), two figure show the message exchange process. The first figure shows the process of heat sink sending absorption message to candidate heat center. The second figure shows the process of heat center sending emission message to heat sink. *ha* denotes heat absorption message and *he* denotes heat emission message.

The key contribution of the proposed method is the development of a heat-passing framework which efficiently interprets the noisy raw data in a robust manner. There is no need to specify the number of clusters for the data and the heat passing process is driven by a set of proposed definitions (heat center, heat sink, heat absorption and emission, and average temperature). Fig. 1 conveys a conceptual idea of the heat-passing framework. Fig. 1(A) indicates that heat is distributed throughout the network with various degrees of efficiency depending on where the heat source is applied. The average temperature (indicated on the top of each figure) of all of the nodes quantitatively reflects the efficiency of heat flowing across the network. We want to determine the optimal nodes (heat centers) that achieve an efficient flow of heat across the whole network Fig. 1(A)(6). The heat-passing process, illustrated in Fig. 1(B), demonstrates the procedure of finding the heat centers and their corresponding heat sinks. We will discuss the process below.

## Methods

Heat-passing framework is to understand the system behavior of a network reflected by the flow of heat over it. Since heat flows through all possible flowing paths between two nodes in a network, if a node has a number of paths to other nodes, the heat flows faster and aggregates rapidly across the network. Consequently, the average temperature is higher when the heat is applied at nodes with a large number of paths to the other nodes, as indicated in Fig. 1(A)(6). The heat passing process aims to optimally identify those nodes, which we call heat centers, through which the heat flow over the network is most efficient. We view packets of heat as refined geometric messages allowing us to efficiently describe paths that are constrained by the intrinsic structure of the network [9]. The strength of the heat message viewpoint is derived from its efficient encoding into the *heat kernel* that accounts simultaneously for all ‘dynamic packets of evidence’ coming from the exchange of heat between *all* pairs of points on the network [12, 13]. For example, the authors in [13] found that heat-diffusion based functional distances among protein domains closely correlate with sequence alignment, structural proximity and phylogenetic similarity. Our proposed framework includes two parts: the definition of the heat flow information on the network and the heat-passing process for the determination of heat centers that facilitate global flow.

### Heat flow on the network

We assume the data can be embedded in a manifold and that the data points are connected by weighted edges that reflect the geometry of this manifold. The heat flow on the manifold can then be quantitatively approximated by the heat kernel *H*_t_(*i*,*j*), normally viewed as a function of two points *i*,*j* on the network at any given time *t*. The physical interpretation of this quantity arises from the application of a unit amount of heat at the node *i*, which is then allowed to flow along the edges. The rate of diffusion over any one of the edges is determined by its weight. The value of the heat kernel *H*_t_(*i*,*j*) is the amount of heat accumulated at *j* after time *t*. The heat kernel is sensitive to the structure of the network as it collects heat based information about all the possible paths between two nodes on the network. The heat will flow faster when applied to a node with many paths to other nodes on the network. We approximate the heat kernel of the manifold using the eigenfunction expansion described below [8, 9]. The network under study is denoted as a graph *G* = (*V,E,W*), where *V* is the set of notes, *E* is a set of edges, and *W* is the weight matrix, so that *W_ij_* is the weight of the edge connecting node *i* and node *j*. Here, we assume that there are no loops in the network, so that *W_ii_* = 0 for each *i*. The graph Laplacian is defined as follows:

L=D−W (1)

where *D* is a diagonal degree matrix and its diagonal entries are given by the summation of rows of *W*:

$$D_{ii}=\sum_{j}W_{ij}$$ (2)

The normalized Laplacian of the graph is defined by

$$L_{N}=D^{-1/2}LD^{-1/2}$$ (3)

Then the heat kernel can be defined by

$$H_{t}(i,j)=\overset{|V|}{\underset{k=1}{\sum }}e^{-\lambda_{k}t}\phi_{k}(i)\phi_{k}(j)$$ (4)

where *λ_k_* is the *k_th_* eigenvalue of the Laplacian and *ϕ_k_* is the *k_th_* normalized eigenfunction. The eigenvalues are ordered so that

$$\lambda_{1}\leq \lambda_{2}\leq \cdots \leq \lambda_{|V|},$$

and we pick an eigenvector for each eigenvalue. If the eigenvalue has geometric multiplicity one, the eigenfunction will be well-defined up to a scalar. The normalization of the eigenfunctions here refers to a choice of constants so that

$$\sum_{i}{|\phi_{k}(i)|}^{2}=1$$ (5)

The quantity *H*_t_(*i*,*j*), the *heat affinity* between the pair of points *i* and *j*, is a measure of heat transfer between the two points after time *t*. We observe the symmetry:

$$H_{t}(i,j)=H_{t}(j,i),$$ (6)

reflecting the symmetry of the Laplacian, whereby the eigenvalues are guaranteed to be real and endowed with a complete basis of eigenvectors.

The average temperature of the whole network when heat is applied on the node *i*, reflects the average amount of heat received by the nodes other than *i* after time *t*. The larger the value, the faster the heat dissipates across the network. The average temperature naturally evaluates the influence of a node, which means, how important the node is among all of the nodes in the network. We therefore use the average temperature to naturally initialize the clustering center preference value of a node (see section 2.2 for details).

$$Temp_{t}(i)=\sum_{j,j\neq i}H_{t}(i,j)$$ (7)

The heat kernel measures the heat affinity between any pair of nodes on the network, which, in turn, reflects how heat flows across the network over time when starting from any initial distribution of heat. The efficiency of heat diffusing over the network depends on the nodes the heat is applied to. We are interested in globally locating the optimal nodes of application of heat to achieve the most efficient heat flow. The optimization problem can be viewed as searching over configurations of the labels g = (g_1_,…,g_*N*_) so as to minimize the energy $E(g)=-\sum_{i=1}^{N}H_{t}(i,g_{i})$. It is more convenient to think of maximizing the net similarity, which is the negative energy [10].

$$g=\underset{g}{\mathrm{argmax}}(\sum_{i=1}^{N}H_{t}(i,g_{i})-\sum_{i=1}^{N}\chi (g_{i}))$$ (8)

where *N* is total number of points, *t* denotes a specific time scale and *g_i_* denotes the label for point *i*. The first term is the summary of the heat affinity within each cluster and the second term is introduced to make sure that if a node is voted as an exemplar by other nodes, it has to be its own exemplar. The *χ*(g_*i*_) is set to ∞ if node g_*i*_ does not select itself as its own exemplar and 0 otherwise [14]. Note that, a trivial solution for this optimization problem is that every node selects itself as its own exemplar. To avoid this trivial solution, an addition input cost is introduced while making a point into an exemplar [14, 15]. The heat center preference value explained below is one of solution to avoid the trivial solution as suggested in [14].

As shown in [10], an optimization problem of this kind can be solved by the message-passing algorithm. We adapt this algorithm to solve Equation 8. Beyond solving this optimization problem, we develop a number of other physics based concepts associated with heat flow over network. We describe these principles within a heat-passing framework below.

The calculation of the exact heat kernel is computationally expensive as calculating the entire spectral decomposition of the normalized Laplacian *L* has complexity *O(n^3^)* using QR algorithm. This can be reduced to *O(n^2^)* as graphs are generally locally connected and hence sparse using the more efficient Lanczos method [16]. The complexity can be further reduced to *O(kn)* by trading off accuracy and calculating only the first (smallest) k(k≪n) eigenvectors and eigenvalues and approximating the summand in Equation 4 using these values. The error bound is (generally) linear in 1/*k* as larger eigenvalues correspond to local frequencies or oscillations (intra-cluster) giving little information about community structure or data relationship (inter-cluster) [17]. However, we perform the spectral decomposition only once and exponentially re-weight the eigenvalues in the summand for any value of the time parameter *t*.

### Heat passing process

As mentioned, the heat passing process aims to seek out the optimal heat centers and corresponding heat sinks by which the heat diffusion over the network is most efficient. The whole process is driven by the heat flow message without requiring any prior knowledge about the data. Even the number of optimal heat centers is revealed as a *result* of the heat-passing procedure. Two message packets are defined for the heat-passing procedure (see Fig. 1): heat emission *he*(*s,c*) and heat absorption *ha*(*s,c*). The heat emission message *he*(*s,c*), sent from candidate heat center *c* to its potential heat sink *s*, reflects how well the center *c* transmits heat to sink *s* by taking into account the support from the other sinks of the *c*. The heat absorption message *ha*(*s,c*), sent from the potential heat sink *s* to heat center *c*, reflects how well the sink *s* absorbs the heat from the center *c* by comparing it to the heat absorbed from other competing heat centers.

We can initialize each node with a heat center preference value *hcpv*, reflecting how well the node prefers itself to be a heat center. The heat center preference value plays an important role in the initialization of the heat passing process for the determination of heat centers. We use the average temperature, defined in Equation 7, as the heat center preference value of a node. This is because the higher the average temperature, the more the node emits heat to other nodes, therefore, the better it prefers to be the heat center.

We initialize the heat emissions to zero: *he*_0_ (*s,c*) = 0 because every node is treated equally at the begin. The heat absorption for each node is initialized as follows:

$$ha_{0}(s,c)≔H_{t}(s,c)-\max_{c^{\prime }\neq c}H_{t}(s,c^{\prime }).$$ (9)

After the first calculation, we iterate the process and modify the heat absorption.

$$ha(s,c)≔H_{t}(s,c)-\max_{c^{\prime }\neq c}(he(s,c^{\prime })+H_{t}(s,c^{\prime })),$$ (10)

where the heat emission *he*(*s,c)* of *c* with respect to *s* is defined iteratively by

$$he(s,c)≔\min\left\{0,ha(c,c)+\sum_{s^{\prime }\notin \{s,c\}}\max\{0,ha(s,c)\}\right\}.$$ (11)

The self-heat absorption *ha*(*c,c*) is set to the heat center preference *hcpv* minus the largest heat affinity between the node *c* and all other candidate heat centers. In addition, the self-heat emission *he*(*c,c*) is defined iteratively by

$$he(c,c):=\sum_{s\neq c}max\{0,ha(s,c)\}$$ (12)

During the iteration, the heat emission and absorption is dynamically updated to reflect the accumulated evidence for a node be a heat center as well as the evidence for a node be the heat sink for some other specific heat center. In later iterations, according the definition above, the self-heat absorption for some nodes can become negative, which means those nodes would prefer to receive the heat from another node rather than themselves. Consequently, as described in Equation 11, the heat emission *he*(*s,c*) for some nodes will drop below zero when they are assigned to the other heat centers. Therefore, those nodes will lose the competition to be a heat center as only the maximum heat emission is considered for competing, as described in Equation 10.

The normalized heat transfer coefficient *ht*(*s,c*) is then defined at each stage by

ht(s,c)≔he(s,c)+ha(s,c) (13)

For any node *c*, its heat sinks set *S_c_* is defined by

$$s\in S_{c}\Leftrightarrow ht(s,c)=\max_{z}ht(s,z)$$ (14)

We say the *c* is a heat center for the sink *s* if $s\in S_{c}$. The iteration process stops after a fixed number of iterations or after the changes in the messages falls below a threshold.

In general, the heat passing process includes two alternative processes (see Fig. 1(B(1)) and 1(B(2))): the heat sink searching for its appropriate heat center and the heat center searching for its appropriate heat sinks. The first process is initialized by a heat sink to show how it thinks the candidate heat center is the optimal one after comparing the candidate heat center to all other possible heat centers. The heat absorption message sent from the sink to the candidate indicates how well the sink absorbs the heat from the candidate heat center by comparing it to the heat absorbed from the other competing heat centers. The second process is initialized by the candidate center to show how suitable it is as a center for the heat sink after collecting the supporting evidence from its other heat sinks. The heat emission message sent from the heat center to the sink indicates how well the center sends the heat to the sink by taking into account the support from the other sinks of this center. These two processes iteratively determine the optimal heat centers and corresponding sink sets. This is enabled by maximizing normalized heat transfer coefficient (Equation 13) when each pair of heat center and sink are optimally matched. Note that, the optimum heat center may not align well with the average center of nodes because 1) heat-passing clusters the nodes based their connectivity which might not consistent with average center determined by compactness, 2) the optimum heat centers have to be actual nodes picked up from the data members while the average central nodes dose not necessarily lie on the actual nodes.

### Data interpretations revealed by heat-passing

Bringing raw data to the stage of meaningful interpretation involves at least three main tasks: ranking, visualizing, and clustering data. We will show that the heat-passing framework can complete those three tasks for all data at once. We define the heat distance, heat coordinates and heat centers and sinks with respect to ranking, visualizing and clustering.

#### 1. Heat distance

The heat distance can be defined from the heat kernel Equation 4 as follows [13]:

$$D_{t}(i,j)=H_{t}(i,i)+H_{t}(j,j)-2^{*}H_{t}(i,j)$$ (15)

Given a query data, we can rank the data points according their heat distance to the query. If we pre-compute heat distance, one can efficiently search the database for a given query.

#### 2. Heat coordinate

The heat coordinate can also be derived from the heat kernel as follows:

$${\mathrm{Cor}}_{t}(i)=(e^{-\lambda_{1}t∕2}\phi_{1}(i),e^{-\lambda_{2}t∕2}\phi_{2}(i),\ldots ,e^{-\lambda_{k}t∕2}\phi_{k}(i))$$ (16)

Here, *λ_k_* is the *k_th_* eigenvalue of the Laplacian and *ϕ_k_* is the *k*-th normalized eigenfunction. The heat coordinate is defined on a space of dimension *k* (less than the number of the data points). Typically, the *i*-th coordinate will decay faster than the *j*-th coordinate when *i>j*, so that a choice of *k* corresponds to the retainment of *k* significant terms in the heat coordinates.

#### 3. Heat center and sink

As mentioned already, the data-driven heat-passing procedure reveals optimal heat centers together with their corresponding regions of influence consisting of optimal heat sinks for each heat center. One gets thereby, a natural partition of the nodes into different clusters with the heat centers becoming the cluster centers and the corresponding heat sinks the cluster members. In a precise form, for each heat center *hc*, its region cluster *S_hc_* is defined by

$$x\in S_{hc}\Leftrightarrow ht(x,hc)=\max_{z}ht(x,z)$$ (17)

where *z* denotes competing heat centers and *ht*(*x,hc*) denotes the normalized heat transfer coefficient between *x* and *hc*.

### Performance index

We define three performance indices, accuracy index (AI), rand index (RI) and quality index (QI), to evaluate the performance of our method [18]. The index values are normalized to [0, 1]. The larger the value, the better the performance is. The first two indices are used to evaluate the accuracy of the corrected assignment. The third index is used to evaluate the quality of the cluster assignment in terms of the compactness of the intra-clusters and spareness of the inter-clusters.

#### 1. Accuracy index and Rand index

The accuracy index is simply calculated as the ratio of the number of images with a correctly assigned class and the total number of images. A more advanced index for accuracy evaluation is rand index which evaluates the match between system-generated clusters to the ground truth. If C is the number of correct match and I is the number of incorrect match, RI is defined as:

$$RI=\frac{C}{C+I}$$ (18)

Generally, we compare all pairs of data after clustering. We consider a pair to be a correctly matched pair if the pair of data is in the same cluster in both the ground truth and the system-generated clustering, or if they are in different clusters in both the ground truth clustering and the system-generated clustering. We consider all other cases to be incorrect match. The value of RI falls in the range of [0, 1]. The data perfectly matches the ground truth if RI is 1.

#### 2. Quality index

The widely-used silhouette index is chosen as quality index evaluate the quality of the cluster assignment in this work. Equation 19 shows the formula for computing the silhouette value.

$$QI(K)=\frac{1}{N_{k}}\sum_{i=1}^{N_{k}}\frac{b(i)-a(i)}{\max\{a(i),b(i)\}}$$ (19)

where, *QI*(*K*) denotes value for the clustering with *K* clusters, *N_k_* denotes the total number of samples, i denotes the *ith* sample, *a*(*i*) denotes the average similarity of sample i to all other samples in the same cluster, and *b*(*i*) is the minimum value of the set of all the average similarity scores from sample i to all other clusters. The range of *QI* value is from −1 to 1, with 1 representing perfect clustering, 0 representing random clustering, and −1 representing perfect anti-clustering. A QI value greater than 0.5 indicates a good clustering, and less than 0.2 indicates a lack of substantial cluster structure. Note that we normalize the QI to the range [0, 1] in our experiment.

## Results

In this section, we have carried out several sets of experiments in partitioning complex network into a small number of clusters, clustering, ranking and visualizing the Globin protein family into biological meaningful subfamilies and determining the conformational states of macromolecules in the presence of a high level of noise. Our experience for setting time parameter is that a range of small diffusion time provides a high resolution view of the network and is thus suitable for analysis. In experimental tests below, we set time parameter *t* of 4 and max iteration of 20000 for heat passing process.

### Community Structure Detection

Recently, we have seen an exponential growth of networks in social science, economics, and communication with the advancement in technology. It is of great interest to develop approaches to reduce the complexity for better understanding the networks. These needs have resulted in research efforts such as partitioning the large network into a number of small subnetworks. However, many challenges remain in automatically partitioning the network with communities overlapping and large portions of missing and spurious links [19–21]. In this experiment, we will demonstrate the heat-passing is able to solve the detections of the community structure in a robust manner without depending on any prior knowledge about the network, including the number of the communities.

**Figure 2:**
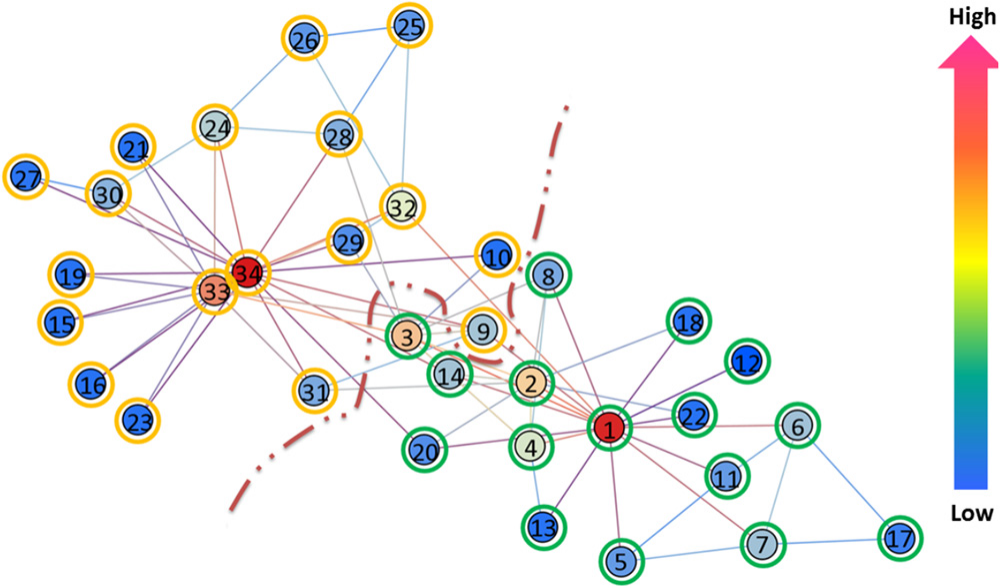
Community structure detection in Zachary’s karate club network. Two clusters are revealed by our method. The nodes circled in orange belong to one faction, and the nodes circled in green belong to another one. The color of a node indicates the average temperature of the node. The closer the color is to red, the higher the average temperature, therefore the better the node prefers to be heat center. The closer the color is to blue, the lower the average temperature, therefore the better the node prefers to be heat sink. A color bar is displayed on the right side. We obtained G 1 = {1: 8, 10: 14, 17, 18, 20, 22} and G2 = {9, 15, 16, 19, 21, 23: 34}.

We test a well known network about a karate club, which is constructed by Wayne Zachary after his observation of social interactions among members of a karate club at an American university [22]. The club was split into two smaller clubs because of a conflict over the price of karate lessons that arose between the club’s president and main teacher. The two smaller clubs are then headed by the president and main teacher, who are denoted by node 34 and 1, respectively in Fig. 2. The original network is used as the input to heat passing framework. Fig. 2 shows the partition result. There are two groups revealed by our method (members belong to the president’s club are highlighted by orange circle and members belong to the teacher’s club are highlighted by green circles, as shown in the Figure). Two heat centers are located by our method (person number 34 and 1), which matches the leadership in the actual karate social network. We partition the club into two groups: G1 = {1: 8, 11: 14, 17, 18, 20, 22} and G2 = {9, 10, 15, 16, 19, 21, 23:34}, where 11: 14 denotes the integers in the range of [11, 14] and 23: 34 denotes the integers in the range of [23, 34]. The partition result by our method is consistent with actual observation by Zachary except for the member number 9. In addition, we color the each node according to the average temperature value (see definition in Equation 7). The closer the color is to red, the higher the value of the average temperature, therefore, the larger the value of the heat center preference value of a node. We can clearly observe that node 34 and 1 are very close to the red end.

It is of interest to further investigate the case of member number 9. As stated in [22], member number 9 is a supporter of the president but becomes a member of the teacher’s club after the fission. This is because that he has to join the teacher’s faction for the black belt (master status). However, structurally it is clear that he should be classified as member of president’s faction. It is an overriding interest that leads to his factional affiliation to teacher’s club after the split. Therefore, the classification result by our method is consistent with the actual social structure of the karate network based on the interactions among members. In a recent study [21], the authors propose an efficient method with application to the partition of members in the karate club. Although they demonstrate an impressive partition, the classification of two members (9 and 10) is not consistent with the actual observation by Zachary. We think the case member 9 can be explained by the similar reason above. For the case of member 10, we think it is because the person 10 has connections to members in both factions. However, our heat passing framework captures his/her preference to president’s faction based on the intrinsic relationship to other members. For comparison, we run original affinity prorogation on karate network. We found that AP identified three groups of nodes with cluster centers of 1, 34, and 32. This is inconsistent with actual observations.

### Clustering, ranking and visualizing Globin protein family

Clustering, ranking, and visualizing proteins based on their sequence or structural similarity are useful in assisting biologist to infer the functions of un-annotated proteins [4, 23]. Given query sequence, searching databases of protein sequences or DNA for evolutionary or functional relationships are of great interests for biologists. The classification of a large number of proteins into biological meaningful subgroups would speed up the database searches and improve the inference of protein function. The visualization of protein in a Euclidean space would provide graphical view of the proteins in a relative position, which greatly free biologist’s focus on data process but the analysis. We will show how the heat-passing is able to cluster,rank, visualize the proteins at once.

**Figure 3:**
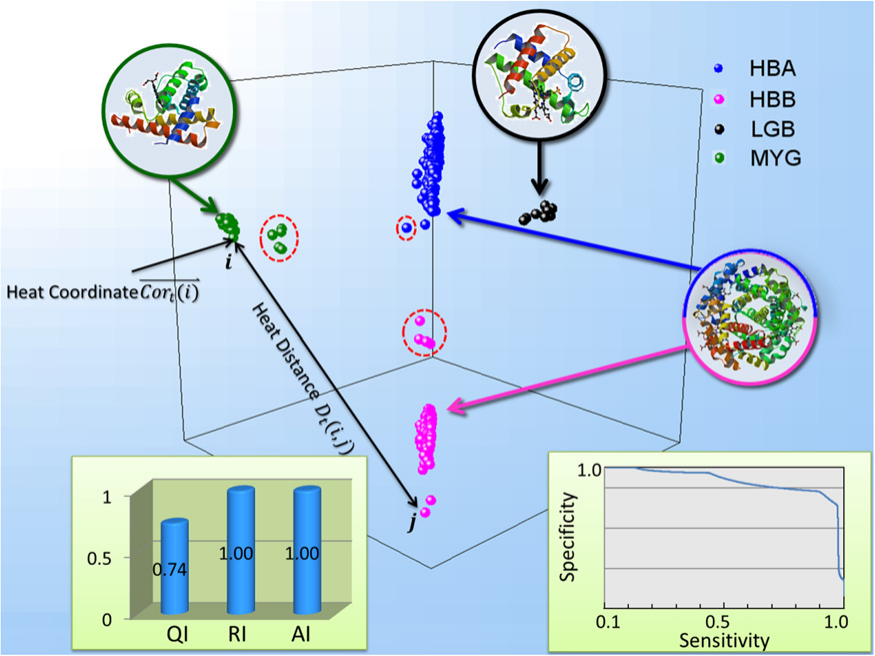
Test for clustering, ranking and clustering globin protein. Figure displays test result for the analysis of globin proteins. The 519 proteins from globin family are plotted in the 3D Euclidean space according to their heat coordinate *Cor^t^*. Different clusters are colored differently: Blue points are *α*-globin protein, purple points are *β*-globin protein, black points are leghemoglobins and green points are myoglobins. The performance index for clustering are listed on the bottom left with RI and AI being 1.00 and QI being 0.74. The specificity-sensitivity curve on the bottom right is used to evaluate the performance ranking and searching based on the heat distance. The PDBID for three proteins used to display 3D structure are 1FSX (*α*-globin and *β*-globin), 1B1N (leghemoglobins), 2MM1 (Myoglobins).

We demonstrated the feasibility of heat passing approach in the context of analysis of the globin proteins family, which contains a large family of heme-containing proteins functioned in binding and/or transportation of oxygen. We used a number 519 (out of 630) globin sequences released with HMMER2 software package [24, 25]. The selection includes four types of chains, a number of 235 *α*-globin (HBA) subfamilies, 197 *β*-globin (HBB) subfamilies, 17 Leghemoglobin (LGB) subfamilies and 70 Myoglobin (MYG) subfamilies according to the SwissProt database annotation [26]. The BIOCONDUCTOR package PAIRSEQSIM is used to compute the distance matrix among the all pairs of proteins (519*519) [27]. We show the testing result in terms of clustering, ranking, and visualizing in the Fig. 3. The clustering performance is evaluated by the AI, RI and QI, which are 1.00, 1.00 and 0.74 respectively. Heat-passing reveals four subfamilies with a perfect classification according to the performance index. We then show the protein ranking by specificity-sensitivity curve according to the heat distance defined by heat-passing. We find that heat-passing performs exceptionally well on the ranking and searching according to the specificity-sensitivity curve. Furthermore, the figure displays a visualization of the sequence space of 519 globin proteins. For illustration purposes, we only choose the first three heat coordinates for each proteins. The 3D representation is quite informative and provides a clear picture about the relationship among the proteins. Four classes of the globin family are visually distinguishable based on the heat coordinate of each protein in the embedded space.

It is interesting to further investigate the distribution of the proteins in the embedded space. The outliers of each cluster is of interest because it normally reflects the function difference from the proteins in the main cluster. We first observe that a group of myoglobins (highlighted by red dashed circle) are slightly away from the rest of the myoglobin cluster. We mapped those proteins back to the original sequence file and find that they are all fish proteins: MYG-CYPCA (COMMON CARP), MYG-GALAU (SHARK), MYG-GALJA (SHARK), MYG-HETPO (PORT JACKSON SHARK), MYG-MUSAN (GUMMY SHARK), MYG-THUAL (YELLOWFIN TUNA). The positioning of the protein in the embedded sapce is encouraging because the fish myoglobins are experimentally observed with different structures from the mammalian myoglobins [28]. Positioning the proteins with heat coordinates, we can easily view the structural distinction based on the protein sequence. In addition, we have the observations for other two globins cluters: *α*-globin cluster (HBA) and *β*-globin cluster (HBB) (all highlighted by red dashed circle), for example, HBAM-RANCA and HBB-HETPO. We therefore predict that those globins might be structurally different from the rest of the cluster. We leave the biological verification of this predication in our future work. The group of leghemoglobins is very tightly centered together which is consistent with the observation in [24]. With the only input of protein sequence similarity input, heat-passing clearly interprets the proteins of globin family. We can easily tell the structural and functional relationship within a subfamily and across different types of subfamilies through the global picture about the data.

**Figure 4:**
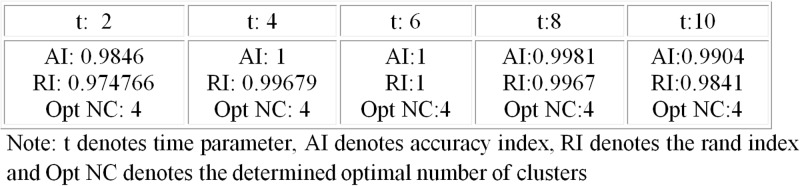
Clustering of globin protein data at different time parameter. Table shows the clustering performance of heat passing with different time parameter from 2 to 10.

One critical parameter for the heat passing that has not been fully discussed in the experiment is the heat dissipation time *t*. The time parameter *t* is important as it essentially reflects the resolution of the network. The larger the time, the more globally the structure is exploited. While we understand that there is no unique value of time that best describes the network, based on the experiments, heat passing prefers to a range of small diffusion time provides a high resolution view of the network and is thus suitable for analysis of underlying structure of a network. In this test, we select the time *t* from 2 to 10. The clustering performance can be found in the Fig. 4. As we can see from the table, the clustering performance is consistently good with a wide range of time *t*.

In addition, for comparison we run original affinity prorogation on globin protein network, we found that AP identified 22 groups of proteins while the correct number is 4. The *AI* and *RI* for AP are 0.47 and 0.78 respectively, which are lower compared to heat passing (*AI*:1.00 and *RI*:1.00).

### Macromolecular heterogeneity

Conformational heterogeneity presents a significant challenge in determination of high resolution 3-D structure using single particle cryo-EM [29–31]. The structural heterogeneity, stemming from either varying compositions or varying conformations, will lead to a combination of a mixture of images from particles a different structure and result in a smeared or even incorrect reconstruction, if not taken into account. A noise-robust (the image is extremely noisy), assumption-free (the number of the conformational states is unknown) clustering approach would pose a clear advantage over the existing methods. In this test, each projection image is a node in the network and the the similarity between a pair of images is assigned to the edge weight. The similarity between a pair of projection images is computed based on Fourier slice theorem [32, 33]. We firstly introduce the details about how to compute the similarity between projections image and build the network for the images, and then how the heat-passing method solve this challenging problem below.

**Figure 5:**
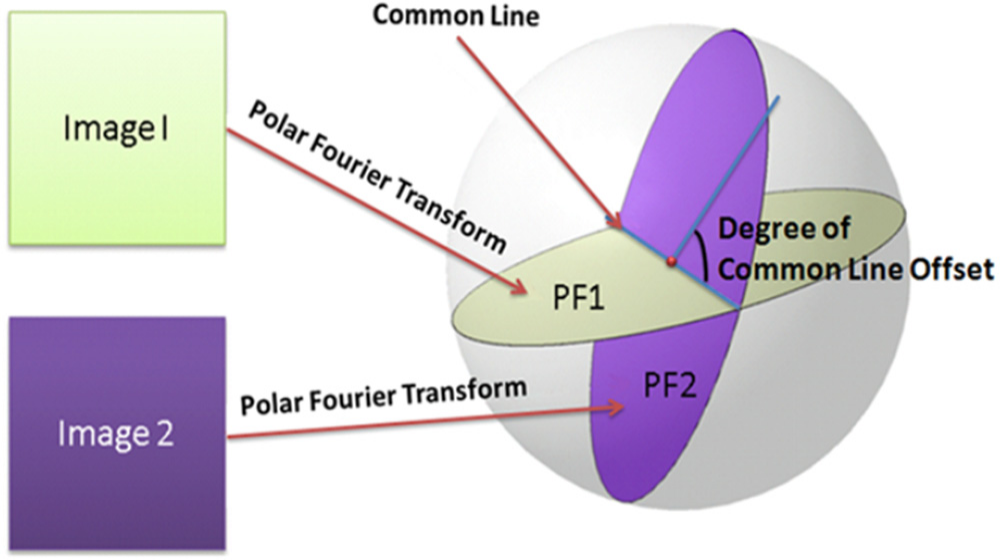
Illustration for Fourier slice theorem. Figure illustrates the Fourier slice theorem about computing the common line between two projection images.

**Similarity calculation among the projections images**. According to the Fourier Central Section theorem, the 2-D Fourier transform of a projection image is equal to a slice through the origin of the 3-D Fourier transform of the corresponding 3-D object. Therefore, the Fourier transforms of every pair of projection images from the same object have an intersection line (common line) Fig. 5. The Fourier values along the common line from those pairs of projection images should be identical. Since the views of the images are not known, we first compute the similarity scores between all line pairs (at a given angular sampling step size) of two images and then set at the best similarity score. At a given level of noises and discretization errors, the higher the similarity score is, the greater is the probability that two images are from particles of the same conformation. The steps for computing the similarity between a pair of images are summarized as follows:

1. Compute the Polar Fourier Transform (PF) of each image using a constant angular sampling (In this study, we used 1.8 degrees which results in 200 lines for each image).
2. Compute the similarity scores for all line-to-line pairs between the two images (In this study, we obtain 200 x 200 scores for a pair of images).
3. Choose the largest similarity score to be the distance between two images. The similarity is computed as follows:
  $$D\left(A,B\right)=\frac{1}{N}|\sum^{\text{​}}\vec{F_{A}}\left(i\right)-\vec{F_{B}}\left(i)\right|$$ (20)
  Where *D*(*A*,*B*) denotes the distance between the common lines from image *A* and image *B*, *N* denotes the number of the sample points along the common line, and $\vec{F_{A}}(i)$ and $\vec{F_{B}}(i)$ denote the Fourier value for *ith* sample point on the common lines from image *A* and *B* respectively.

**Figure 6:**
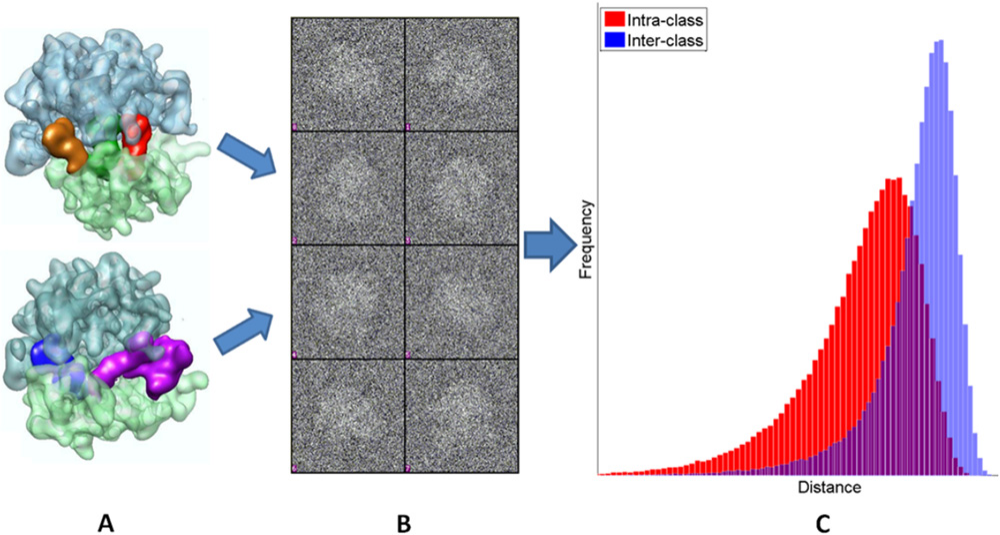
Illustration for two ribosome conformational states, projection images and distance histogram. Figure(A) shows the 3D structure of two 70S ribosome conformation states: the unratcheted state in complex with three tRNAs (orange, green, red) and the ratcheted state in complex with one tRNA (blue) and EF-G (purple). (B) The mixture of the projection images from two states at SNR = 0.1. (C) The histogram of the common line based pair-wise distances for intra-class (red) and inter-class (blue). The distance for images across different classes is larger but significantly overlapped with intra-class distance because of the existence of noise.

**Test on ribosome data with conformational states**. Test on ribosome data with two conformational states, The 70S E. coli ribosome in the process of translation is a well-known system with heterogeneous conformations in the solution. The subtle structural differences among the different conformations pose a great challenge in determining the number of states and accurately classifying the particles into appropriate states. In our experiments, the 3-D density maps of the two distinct ribosome states (Fig. 6(A)) are provided by Sjors H W Scheres [34]. The projection images are simulated by projecting the ribosome structures at random views, corrupted by zero-mean Gaussian noises at different levels, and then randomly shift the center of the images (up to %6 of image size). Fig. 6(B) illustrates the the projection images of two ribosome molecules with noise (SNR = 0.1). When SNR is at 0.1 or lower, the ribosome particles are barely visible, and the visual contrasts of these images are similar to or lower than typical single particle cryo-EM images. We performed the clustering experiment with two distinct ribosome states (states I and II). The first conformation (I) is in un-ratcheted state with three bound tRNAs at the A, P, E sites but without bound elongation factor G (EF-G). The second conformation (II) is in a ratcheted state with bound EF-G and a single tRNA in the hybrid P/E position. The structural difference between these two states, mainly caused by the ratcheting of a 30S subunit relative to the 50S subunit and by the bound tRNAs and EF-G, are recognizable in the surface views of the established structures (Fig. 6(A)). Four hundred projection images (130x130 pixels) at random views were generated for each of the two ribosome conformations. Noise was then added to the projection images to obtain test image datasets at multiple SNRs (range from 0.05 to 5). The distance score for all image pairs, including images from the same conformational state and from different states, is first computed. The histograms for intra-class and inter-class distance scores is significantly overlapped when SNR is low (0.1 for example Fig. 6(C)), which indicates there is a significant overlapping between two clusters of images. As expected, the clustering became more challenging at low SNRs.

**Figure 7:**
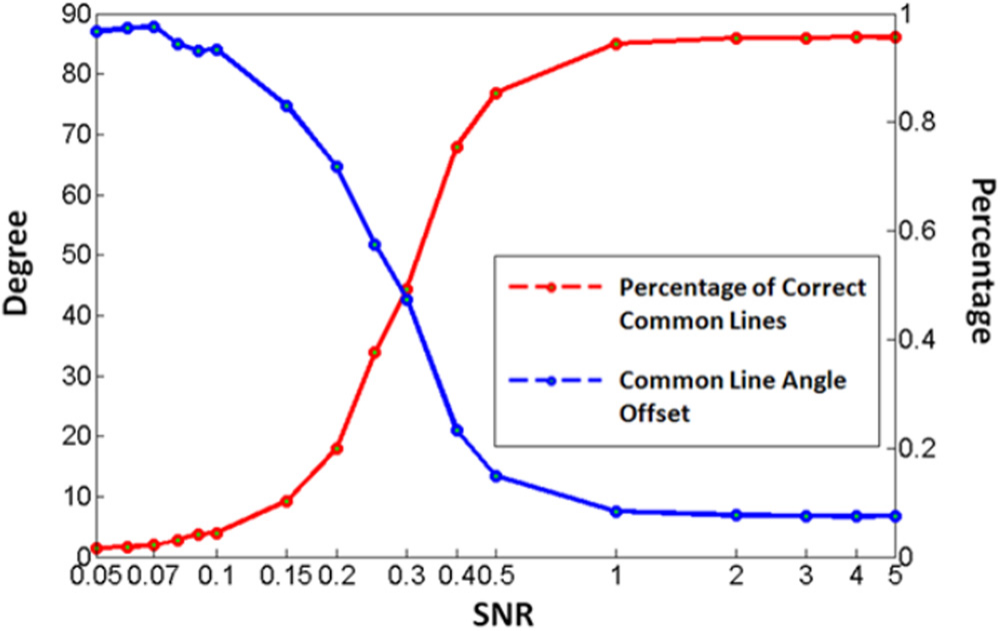
Illustration for common line angle offset. The degree of common line offset reflects the error angle between the detected common line and the real common line because of the noise. Figure plots the common line angle offset and percentage of correctly detected common lines at different SNR levels. As we expect, the value of the degree is decreasing and the percentage is increasing with respect to the increasing value of SNR.

In addition, the common line measurement is historically sensitive to noise. We demonstrate that the common line position error and the total percentage of corrected measured common lines. The correct positions (ground truth) for the common lines are calculated based on the projection angles. The common line position errors are calculated according to the difference between ground truth positions and the measured positions under different SNR values. An average line position error was calculated for all pairs of images for each SNR level. When the signal is strong, the error should be nearly zero. However, the line position error is expected to reach 90 degrees if the noise level is too high and the measured line positions approach random positions. Similarly, we also recorded the percentage of the correct (error less than 5 degree) common line positions for each noise level. The results were displayed in Fig. 7. As expected, the common line position errors become smaller and the percentage of correctly identified common lines increase as SNR increases.

**Figure 8:**
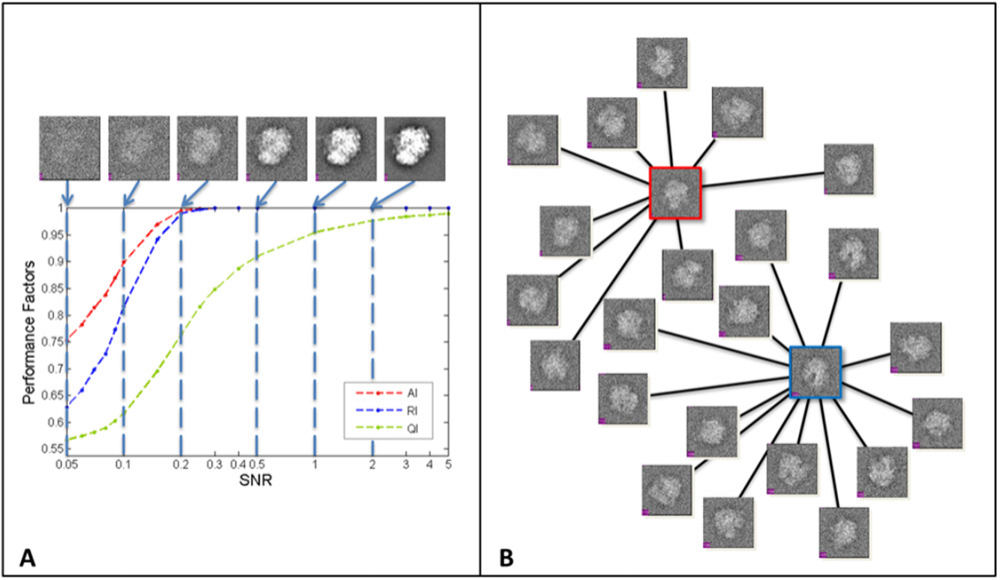
Performance evaluation for ribosome test. Figure (A) shows the performance index for all of the raw images being classified into two clusters. On the top of the figure are the projection images at corresponding noise levels. The plot illustrates the value of AI, RI and QI with respect to the increasing value of SNR. Figure(B) displays two images (boxed in red and blue colors) for the heat centers and their own images for heat sinks at SNR = 0.2.

The clustering results on these test images are shown in Fig. 8. In this test, the optimal number of classes (i.e. conformations) (K = 2) is correctly determined even when the SNR decreases to 0.05. Three performance index for different levels of noise are plotted in Fig. 8(A). As expected, the performance index increases with the increasing SNR. Importantly, the clustering maintains a perfect accuracy and rand index at SNR as low as 0.2, a noise level at which the image contrast is comparable to that of actual cryo-EM images. When SNR is at 0.2, our method is able to accurately classify 99% percent of images even in the presence of such overwhelming level of noises. The clustering accuracy drops as SNR decreases but remains reasonable (AI = 0.75 and RI = 0.63) even when SNR reaches 0.05. With SNR equal to 0.05, one can hardly recognize the existence of a ribosome particle in the image box, and the image contrast at this SNR level is probably worse than that of most real cryo-EM images. The Fig. 8(B) displays that two revealed images (boxed in red and blue colors respectively) for heat centers which link to their own images for sinks (images are displayed at SNR = 0.20). These results demonstrate that our method is very robust to high noise levels. Since the similarities among images are computed based on Fourier Central Section Theorem, a measurement that only uses a small fraction of the image data (i.e. a line in a 2-D plane) and is known to be sensitive to noises [32], these results are very exciting in a sense that a well documented issue can be partially overcome by our heat-passing framework. Furthermore, according to Fig. 8(A), comparing the SNR dependence of QI, AI and RI, we find that QI is positively correlated to increased AI and RI. This observation suggests that QI can be used in evaluating the confidence level of the clustering result. This is critical in practice for real cryo-EM projects since neither the actual number of classes nor the correct class member assignment is known.

**Figure 9:**
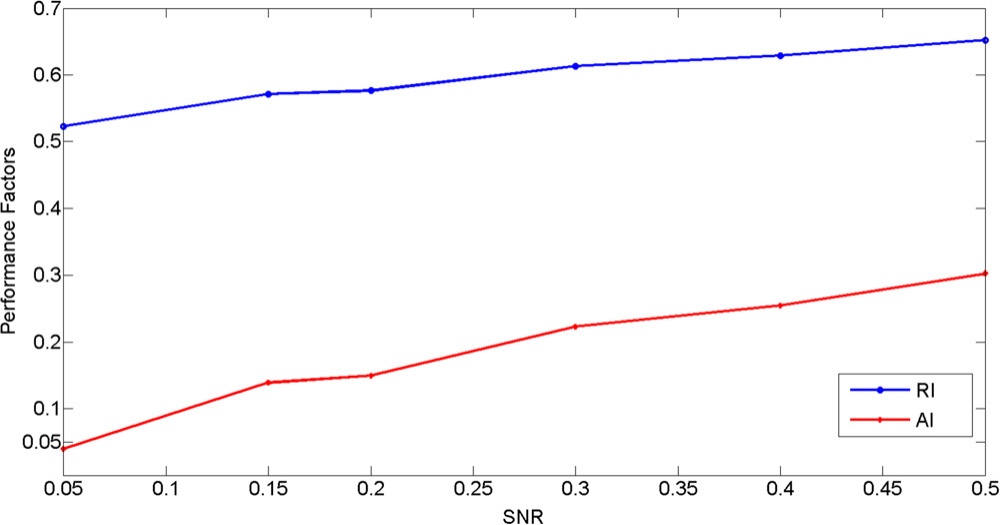
Evaluation of affinity propagation in ribosome test. Figure shows the performance index for all of the raw images being classified into two clusters. The plot illustrates the value of AI and RI with respect to the increasing value of SNR.

As heat passing is derived from affinity propagation based on heat kernel, we compared their performance for clustering ribosome images from two conformational states. We used the same image data sets above for affinity propagation. In Fig. 9, we can find that Affinity propagation consistently performs worse than heat passing with lower values of AI and RI. Another interesting observation is that the AI for affinity propagation is very low with the value under 0.3 even when SNR increases to 0.5. And the AI is close to 1.0 for heat passing when SNR is near to 0.2. This can be explained by the fact that Affinity propagation is not able to identify the correct number of clusters. The identified clusters number is 15, 7, 8, 9, 13, 14 for the SNR from 0.05 to 0.5. Since the AI is sensitive to the ground truth label, it stays low for all of the tests. In contrast, the heat passing is able to identify the correct number of the clusters and both AI and RI remain higher for all of SNR values.

## Discussion

The abundance of newly-measured data from various disciplines, such as social networks, the internet, biological networks, experimental measurements, and multimedia, has shifted scientists’ attention from data collection to data analysis. Despite the broad success of current approaches for data analysis, heterogeneity, incompleteness, measurement error, and inherent noise are sources of concern challenging any claim of complete data interpretation. In the previous sections, we have described a new heat-passing framework for exploiting the intrinsic similarity relationship behind a network of noisy and incomplete raw data that maps the network out in a robust manner. We discuss the features of the heat-passing framework and our contributions as follows:

### Heat-passing provides a robust, global and structure-based method for data interpretation

A good data analysis approach should be robust to noise, as the observed data, especially biological data, is often noisy and heterogeneous with a low coverage of the whole system. Any approach, if simply based on individual local pairs of data or a small number of neighbors, is likely to be overwhelmed by the noise and incompleteness. It is of great importance to place all of the data in a network and study the behavior of the network system governed by agreement among all individual interactions. The desired effect is to generate and display significant knowledge of the overall global structure of the data. Heat-passing is a type of structure-based approach, with the heat flow encoded as structure-aware information that enables diffusion across the network to reveal the intrinsic relationship among the data points. The excellent robustness against noise is highlighted by its good performance in resolving the conformational states for the macromolecular at noise level (*SNR* = 0.05).

### Heat-passing is independent of prior knowledge about data

In addition to being robust to noise, the dependence on prior knowledge about data is also crucial for a computational data analysis approach. The importance is particularly highlighted when scientists have limited knowledge or none about the data, for example, when a new biological machine is firstly studied. Any prior assumption applied to such data on the basis of previous experimental results or observations, is likely to bias investigation. Given the abundance of instances where only little a priori knowledge about the data is available, we must favor approaches that are independent of prior knowledge, which can provide the insight about the data with little or no human intervention. Heat-passing provides a coherent framework for learning from data and for reasoning about underlying structure without depending on much prior knowledge. For example, rather than requiring the number of heat centers to be pre-specified, heat-passing only takes geometric similarity within the data set as input in order to optimally determine the number and location of heat centers as well as corresponding regions of influence. Even though the method is automatic, the process is able to unearth hidden structure from the statistical regularities of large data sets.

### Limitations and future directions

With rapid advances in measurement technology, almost every type of data is accumulating at a rapid pace, and brings to focus robust and intelligent approaches to data analysis. The opportunity is clear for computational scientists to speed up the development of methodology to meet the needs of this ever-growing data. Our heat-passing framework is inspired by the physical process of heat flow. We have outlined its many appealing features and diverse potential applications. We expect heat-passing to be broadly applicable to various areas of data interpretation, and especially as an approach to complex network analysis. However, this paper presents only a first implementation, with very promising results in the completed tests. One area that particularly calls for more investigation is the heat evolution time. Although it is difficult to choose a proper time parameter, it plays a critical role in looking through the geometric structure at multiple scales. In our future work, we will investigate good strategies to determine time as a means for taking stock of the raw data at different level of details.
